# Prescribers’ views and experiences of assessing the appropriateness of prescribed medications in a specialist addiction service

**DOI:** 10.1007/s11096-017-0541-4

**Published:** 2017-10-26

**Authors:** Adejoke Obirenjeyi Oluyase, Duncan Raistrick, Elizabeth Hughes, Charlie Lloyd

**Affiliations:** 1 Department of Health Sciences, University of York, Room 208b, Area 4 ARRC Building, Heslington, York, YO10 5DD UK; 2 School of Health and Human Sciences, University of Huddersfield, Harold Wilson Building, Queensgate, Huddersfield, HD1 3DH UK; 3 Leeds and York Partnership NHS Foundation Trust, 19 Springfield Mount, Leeds, LS2 9NG UK

**Keywords:** Appropriateness, Medications, Medical prescribers, Nurse prescribers, Specialist addiction service, United Kingdom

## Abstract

*Background* Mental and physical health problems are common in people with substance misuse problems and medications are often required in their management. Given the extent of prescribing for service users who attend specialist addiction services, it is important to consider how prescribers in this setting assess the appropriateness of service users’ prescribed medications. *Objective* To explore prescribers’ views and experiences of assessing the appropriateness of medications prescribed for service users coming in for treatment as well as the differences between prescribers. *Setting* A specialist addiction service in the North of England. *Method* A phenomenological approach was adopted. Semi-structured interviews were conducted with four nurse prescribers and eight doctors. Data were analysed using thematic framework analysis. *Main outcome measure* Prescribers’ views and experiences of assessing the appropriateness of prescribed medications. *Results* Assessment of the appropriateness of prescribed medications involved reviewing medications, assessing risk, history-taking, involvement of service users, and comparing guideline adherence and ‘successful’ prescribing. Doctors and nurse prescribers assessed the appropriateness of medications they considered to be within their competency. Doctors provided support to nurse prescribers and general practitioners (GPs) when dealing with issues around prescribing. *Conclusion* Assessment of the appropriateness of prescribed medications is complex. The recent reduction in medical expertise in specialist addiction services may negatively impact on the clinical management of service users. It appears that there is a need for further training of nurse prescribers and GPs so they can provide optimal care to service users.

**Electronic supplementary material:**

The online version of this article (doi:10.1007/s11096-017-0541-4) contains supplementary material, which is available to authorized users.

## Impacts on practice

- Nurse prescribers and doctors in a specialist addiction service differ in the types of medications they review but appear to be working within their competency.
- Decreasing medical expertise in addictions may pose a threat to quality decision-making by nurse prescribers.
- The decreasing availability of medical expertise in addiction services presents a challenge to the management of complex service users by GPs.
- There is a need to provide training and support to nurse prescribers and GPs on prescribing for people with substance misuse problems, so that they can provide optimal care to specialist addiction service users.

## Introduction

People with substance misuse problems often have co-existing physical and/or mental health conditions [1, 2], and are prescribed a large number of medications which may sometimes not be justified [3]. Service users who seek treatment in specialist addiction clinics are more likely to have higher levels of dependence and complex needs that include social problems, functional impairment, comorbidities and use of multiple medications when compared with those who do not seek help [4, 5]. These complex needs may influence prescribing decisions made for this population [6]. For instance, prescribing may be targeted at maintaining equilibrium in the lives of service users, which may lead to prescribing outside of guideline recommendations. Furthermore, service users may want certain medications such as opioids and benzodiazepines prescribed for non-medical reasons [7, 8].

Opioids used in pain treatment and benzodiazepines for mental health problems have been implicated in the occurrence of adverse events in people with substance misuse problems. Benzodiazepines, antidepressants, antipsychotics and substances such as alcohol have often been found to be used in combination with opioids such as dihydrocodeine and oxycodone in opioid-related overdose and fatalities [9–11]. Antidepressant prescriptions, especially tricyclic antidepressants (hereafter TCAs), have also been linked to heroin overdose [12, 13].

The large number of people entering specialist addiction services with complex needs and multiple prescriptions provides an important opportunity for exploring addiction service prescribers’ views and experiences of assessing the appropriateness of medications prescribed for service users coming in for treatment as well as the differences between the various types of prescribers. Prescribers included in this study were medical and non-medical. The non-medical prescribers (NMPs) were independent nurse prescribers who could assess and also devise a treatment plan that may include prescribing for service users [14]. NMPs prescribe within their areas of competence [15]. For instance, nurse prescribers working in addiction medicine are able to prescribe substitute opioids, relapse prevention medications, medications for detoxification and vitamin supplements.

Assessment of the clinical appropriateness of non-medical prescribing, including nurse prescribing, have concluded that NMPs generally make clinically appropriate prescribing decisions [16, 17]. However, history taking, assessment and diagnosis skills have been highlighted as areas for further attention.

Service users visiting the service could self-refer or be referred from a range of sources such as general practitioners, psychiatrists, hospital, social services, drug services and the criminal justice system. Consequently, this study explored specialist addiction service prescribers’ views and experiences of assessing the appropriateness of medications prescribed by others.

### Aim

This study explored specialist addiction service prescribers’ views and experiences of assessing the appropriateness of medications prescribed for service users coming in for treatment as well as the differences between prescribers. Appropriateness was considered to involve maximising effectiveness, minimising risks and costs, and respecting the patient’s choice [18].

### Ethics approval

The study was approved by the University of York’s Research Governance Committee and the National Research Ethics Service (NRES) Committee Yorkshire & The Humber. Reference 12/YH/0325.

## Methods

### Study design and setting

A phenomenological approach was taken to explore individual views and experiences of assessing the appropriateness of medications prescribed for service users. Semi-structured qualitative interviews were carried out with prescribers comprising nurse prescribers and medical doctors working at the specialist addiction service. This service is located in a city in the North of England and is a statutory NHS specialist service that provides tier 3 level interventions to adults who misuse alcohol and/or drugs. Tier 3 interventions generally involve the provision of care-planned interventions following a comprehensive community-based assessment [19]. One-on-one interviews were used because it would be very difficult to get time-pressed clinicians together for a focus group discussion. In addition, group interviews may be prohibitive for some prescribers.

### Participants

Twelve prescribers took part in this study, comprising four nurse prescribers and eight medical doctors. In line with qualitative research inquiry, the aim of the sampling strategy adopted was to recruit respondents who could provide valuable insight into the topic and also to provide a broad overview of the perspectives of different prescribers. Consequently, all the fourteen prescribers working at the specialist addiction service during the period of this study were provided with the study details by the chief investigator (A. O). This was followed by a meeting with each prescriber to discuss the study in detail after which written informed consent was sought. Twelve of the fourteen eligible prescribers were interviewed. Participants included five females, three of whom were nurse prescribers and two medical doctors and seven males, of whom one was a nurse prescriber and six medical doctors. The medical doctors had different levels of seniority and included one senior house officer (hereafter SHO), one locum doctor, three specialist registrars (hereafter SpR) and three consultant addiction psychiatrists. Generally, the prescribers represented a broad range of qualifications and experience in the addiction field.^1^ Nurse prescribers’ ages ranged from 34 to 55 years while doctors were between 31 and 65 years.

### Data collection

Data were collected by the first author, A. O. All the interviews were conducted at a time convenient for participants at the specialist addiction service and lasted on average 48 min (range 36–74 min). The topic guide was informed by knowledge of the literature on prescribing and advice from the project advisory group (which included one consultant addiction psychiatrist). The topic guide was piloted with a consultant addiction psychiatrist and covered the following areas: definition of inappropriate prescribing, classes of medications assessed and how assessment is carried out. The interviews were audio recorded (with permission) and transcribed verbatim.

### Data analysis

Data were analysed using thematic framework analysis [20]. Familiarisation involved repeated reading of transcripts alongside listening to the audio-recordings and was followed by a period of descriptive and interpretive coding facilitated by Atlas ti (v 6.0). This inductive approach enabled a deeper understanding of the data [21]. As new themes emerged, they were added to the coding framework. Broader themes were subsequently generated and frequently reviewed while comparing data from participants that supported the themes and also looking for explanations of any differences of viewpoints within the data. Numbers rather than names were allocated to participants in order to ensure anonymity and confidentiality. Trustworthiness of the data was ensured through an audit trail kept by A.O which detailed how data were collected, how themes were formed and how decisions were made during the research process. Furthermore, the interpretation of the data was discussed in-depth with two of the authors (C.L and E.H), who reflected on the plausibility of the themes and the depth of the analysis. A. O has a pharmacy background while C. L and E.H have criminology and nursing backgrounds respectively. D.R is a consultant addiction psychiatrist.

## Results

The following themes emerged in response to how prescribers assessed the appropriateness of prescribed medications: review of medications, assessing risk, guideline adherence versus successful prescribing, history-taking and involvement of service users. There were some areas of differences in nurse prescribers and medical doctors’ approaches and also among the different types of medical doctors. These differences are highlighted in the text.

### Review of medications

The classes of medications reviewed varied among prescribers with three of them (all doctors) with the longest years of prescribing experience stating that they reviewed all of service users’ medications for their appropriateness. One of these three prescribers had 41 years of experience in prescribing and made the following statement:

So I’d look at the list of drugs prescribed and see how they matched up to what I thought the person was showing in terms of addiction illness, physical illness and mental illness [P3, consultant].

The remaining prescribers consisting of other doctors and nurse prescribers described a more limited remit. These doctors considered their scope of practice to encompass medications for mental health illnesses, addictions and sometimes opioids for pain relief while nurse prescribers described a focus on medications used for treating addiction problems. This quote captures a nurse prescriber’s view:

So I don’t really see, with psychiatric medication, that that would be within my remit really. If somebody came and they were prescribed 100 mgs of methadone and they couldn’t even open their eyes then, I would be assessing the appropriateness of the dosage and making necessary adjustments to things like that [P10, NP].

Nurse prescribers further described involving doctors at the specialist addiction service or service users’ general practitioners (hereafter GPs) if they had particular concerns about medications. There was an underlying feeling of cautiousness characterised by their perceptions of their competency. This was captured by the quote below:

As I say, if I was particularly concerned about someone’s mood or I have particular concerns about the medication I would defer to a medic. You know, it’s not an area I feel strongly confident on [P6, NP].

Doctors at the specialist addiction service were a valuable source of support to nurse prescribers in prescribing-related issues. There was also particular reliance on the expertise of consultant addiction psychiatrists by both nurse prescribers and doctors who were not consultants. A doctor described contacting a GP concerning an inappropriate medication and the support of her consultant in providing expert advice when needed:

For the example I started with [*patient with schizophrenia on supra-BNF dose of olanzapine*], I wrote to the GP saying, you know, Mr So-and-So is stable and is relatively symptom free on this but I’m worried about this monitoring [*olanzapine monitoring*] but generally if I think something’s really inappropriate and I’m in a position to contact the original prescriber I’ll try to do that, but I’d always discuss a case with my consultant and make a decision about whether or not I need to do something imminently [P12, SHO].

It appears that prescribers at this specialist addiction service provided a ‘safety net’ function to other prescribers such as GPs:

If I find something that’s maybe been overlooked or prescribed wrongly, then I will let the GP know about it [P5, Locum].

I’d probably look at it [*medication appropriateness*] at the initial assessment and if there’s anything that comes up or that was sort of glaringly obvious I’d refer to the GP and ask the GP to review, if they’re prescribing [P11, NP].

Specialist addiction service prescribers further described GPs’ varying responses to the need for review of service users’ medications:

Yeah. that has happened on a couple of times where I’ve written to the GP to ask them to review… there have been a couple of scenarios where I’ve written and the GP hasn’t responded or the GP has written back saying, I don’t feel I’m the best person to do this, would you refer to a specialist service or would you basically will you deal with it [P12, SHO].

They also described sometimes taking over prescribing of psychiatric medications from GPs:

But in general I’d like to take over all of the psychoactive drugs that somebody gets, at least until the point that we’re sure that the drugs are appropriate and we’ve got some sort of stable situation [P3, Consultant].

### Assessing risk

The evaluation of risk is a theme that was highlighted by all prescribers as a means through which they assess the appropriateness of service users’ medications. All the twelve prescribers said they considered the risk posed by a medication. Some of the quotations captured this:

Well if it’s going to do, first of all, less harm than the actual substance, not more harm, so the actual prescription can be worse than doing nothing [P5, locum doctor].

One prescriber described a service user who she felt had an inappropriate and high risk prescription of olanzapine (an antipsychotic). The service user was an elderly man who was being prescribed olanzapine (25 mg) at a dose higher than that stated in the British National Formulary (BNF) without monitoring by a psychiatrist:

I have a patient who has a very old diagnosis of paranoid schizophrenia dating from his late teens, and for this he’s prescribed a very high dose of medication called olanzapine and he’s prescribed over the limit in the BNF and he’s not under the supervision of a specialist. So I would label that as an inappropriate prescription because (a) he’s elderly, which means that he’s more prone to cardiac disease, and the drug can cause diabetes which can lead to heart disease. It can cause arrhythmias, he’s not being monitored regularly with regards to that, and he’s not being monitored with regards to his clinical symptoms, which, are actually, from a psychosis point of view, negligible [P12, SHO].

The SHO described contacting the service user’s GP concerning the antipsychotic medication. His GP refused to alter it due to the service user’s stability on the dose for a prolonged period. The GP and SHO differed in their views concerning the antipsychotic. There was no change made to the antipsychotic.

### Guideline adherence versus successful prescription

The need to assess if prescribing is in line with guidelines was highlighted. Some prescribers further acknowledged that the need to individualise prescribing and ensure optimal functioning may lead to prescribing outside guideline recommendations. The need to consider the context of prescribing was emphasised by a nurse prescriber:

And I think any comment about any prescribing should only be made when you know about the circumstances in which the decision was made. For example, we prescribe very high doses of some drugs, now some people say that you shouldn’t prescribe at those levels, but they are appropriate if you know about the circumstances [P1, NP].

A consultant addiction psychiatrist also expressed similar views and contrasted guideline adherence with successful prescribing:

Prescribing is something of an art as well as a science, so prescribers will sometimes prescribe things that they know are not really indicated but with the aim of achieving a particular goal [P3, Consultant].

### History-taking

All prescribers identified history-taking as a part of their assessment of the appropriateness of service users’ medications. The prescribers described enquiring about service users’ medical and medication history:

Looking at the history of their substance use, history of any physical health problems, mental health history, and current mental state as well so I’d get the full history and I think then you can kind of gauge whether something might be inappropriately prescribed [P11, NP].

Despite prescribers routinely obtaining a medical/medication history from service users, most reiterated that it was not within their remit to explore the appropriateness of all prescribed medications:

…I would, in as much as part of the assessment, I would ask the service user …are they on any medications. If they are, what it is, what dose, what’s it prescribed for and are they taking it. That would be the total sum of my assessment. I wouldn’t move to beyond exploring that condition or whether that was appropriate, I don’t think that’s my place [P6, NP].

All prescribers further described some challenges with self-report when obtaining service users’ histories. These include problems with the reliability of information provided by service users as some of them may withhold information. This may lead to prescribing of unnecessary medications. Prescribers also described service users who do not know details of their medications such as the name and reason for medication use. Some may be cognitively impaired by substances and therefore unable to provide necessary information. Prescribers may have to contact GPs concerning needed information. There was however an acknowledgment that contacting GPs for information was not always routine practice as prescribers tended to rely on information obtained from service users.

### Involvement of service users

This theme was described by all prescribers. It involved discussing with service users in order to understand their views concerning the appropriateness of their prescribed medications:

Well, firstly I discuss with the patient to see what the patient’s view is, and explain what I think, which are the reasons for this inappropriateness [P13, SpR].

Prescribers also highlighted the fact that lack of engagement by service users may affect prescribing decisions. For instance, service users’ medications may need to be stopped due to repeated non-attendance of clinic appointments.

## Discussion

The evidence from this study shows that the assessment of the appropriateness of prescribed medications is a complex judgment. Besides a few more experienced doctors, all other prescribers (doctors and nurse prescribers) tended to review only the subset of medications which they saw as within their competency. It has been recommended that doctors and nurse prescribers adhere to their areas of competency for safe practice [22, 23]. Nurse prescribers and doctors appeared to be working within their competency.

Published evidence suggests non-medical prescribers generally make clinically appropriate prescribing decisions with the need for further improvement in assessment, diagnosis and history-taking skills [16, 17]. Nurse prescribers described referring service users who they had concerns about their medications to doctors at the specialist addiction service or service users’ GPs. Specialist addiction service doctors particularly represented a valuable source of support to nurse prescribers when dealing with issues around prescribing. The more junior doctors (non-consultants) also relied on their senior colleagues, especially consultant addiction psychiatrists, for expert advice on medications. There was further evidence that prescribers were a sort of ‘safety net’ against medication-related risks as they intervened and contacted GPs if they found serious problems with service users’ medications.

Service users pose particular challenges in terms of complexity and risk issues. They often have complex needs including severe comorbid mental and physical health problems [24–29]. In order to meet these needs, Public Health England [23] has recommended that addiction specialist doctors such as consultant psychiatrists work alongside non-medical prescribers and other doctors in a multidisciplinary team. The drug and alcohol treatment system has however undergone some changes in commissioning in recent years. This has involved a move from mainly NHS service provision to a more mixed economy of service providers [23]. These changes have led to a decrease in the number of doctors including consultant addiction psychiatrists in treatment systems [23], with nurses taking on more prescribing roles. Consequently, there is a reduction in the capacity of these new treatment systems for specialist expertise and complex case management.

It appears that there is a possibility of reduction in the quality of prescribing and decision-making as a result of these changes as nurse prescribers and GPs may not have ready access to support and specialist knowledge when required. The potential for specialists to provide clinical supervision that will support nurse prescribers in making clinically appropriate decisions when needed is also hampered. It appears future prescribing practice in alcohol and drug treatment systems will mostly involve nurse prescribers. This raises concerns about the future review practices of psychiatric medications in addiction services if nurse prescribers are not further strengthened to work with service users, including complex clients. In addiction service users, psychiatric comorbidity is highly prevalent [25–28] and medications used in their management have often been implicated in overdose and fatalities [11–13]. Pharmacists’ support could be enlisted to guide prescribing decisions for service users with complex comorbidity. This approach may assist in improving medicines management among service users.

There is the need to equip nurse prescribers to work with service users, especially complex cases. Given that assessment, diagnosis and history-taking skills are pre-requisites for undertaking the nurse prescribing qualification, these skills may well be further developed through training to enable nurse prescribers manage complex service users, especially those with comorbid mental disorders. Practice should include regular supervision of nurse prescribers by an experienced doctor or nurse prescriber to ensure that they are making optimal clinical decisions.

The relationship between healthcare professionals and service users have changed over the years from a predominantly paternalistic model to one in which service users have increasingly become active partners whose views are important [30, 31]. Involving service users assists the prescriber in eliciting their views and is useful in decision-making concerning treatment [32]. There is evidence that building a positive relationship can lead to positive client and treatment outcomes [33]. Despite these potential benefits, prescribers identified problems that may occur when trying to involve service users in decision-making. The quality of information provided by service users may be poor as a result of cognitive impairment or even deliberate withholding of information. When service users are actively misusing substances, prescribers lose access to the most fundamental tool in medicine, the patient’s self-report [34]. While some prescribers described contacting service users’ GPs for further information concerning medications, this was not done by all prescribers.

Depending on information obtained from only service users in assessing appropriateness implies that medications which are potentially inappropriate may not be identified if service users fail to mention them. There is the possibility that different prescribers may go ahead to prescribe undisclosed medications such as multiple central nervous system depressants. In addiction medicine, there should be careful consideration of self-report and collateral information should be sought where possible [34]. Shared medical records [35] and good communication among different service providers are essential in obtaining accurate medical/medication histories and reducing the potential for multiple prescribing, drug interactions, overdose incidents and conflicting treatment plans [34].

The limited applicability of guidelines to service users was also recognised by prescribers. Guidelines often have a disease-specific focus and limited applicability to the varying needs of individual patients [36]. Although prescribing outside guideline recommendations carries its own risks including the potential for greater severity of unwanted side effects [37], there needs to be a weighing of such risks against more pragmatic outcomes that may be of great importance to service users.

### Strengths and limitations

To the knowledge of the authors, this is the first study to explore the views and experiences of specialist addiction service prescribers when assessing the appropriateness of prescribed medications among service users coming to this setting. Owing to the fact that the interviews were conducted with prescribers after they had taken part in an earlier study in which the appropriateness of opioids and psychiatric medications were assessed using a modified form of the Medication Appropriateness Index [38], it is possible that participation in this initial study may have influenced some of their responses to the different areas explored in the interviews. Consequently, prescribers’ responses might be different if they were interviewed before taking part in this initial study.

The findings may lack generalisability to prescribers in other addiction services, especially given the changes that have occurred in drug and alcohol treatment services in the UK. There has been an increase in the number of third sector organisations (non-statutory service providers and the private sector) providing drug and alcohol services. Availability of medical expertise has also diminished in these services. Further research should involve multiple sites (including services run by the NHS and third sector organisations), to establish if the findings of this study are applicable. Given the reducing levels of medical expertise among staff in specialist addiction services, an important area to explore will be the role and scope of nurse prescribers: including their views on the changing drug treatment landscape, management of service users (especially those with complex needs), the support available to nurse prescribers and their training needs. Similarly, there may well be need to interview GPs on these areas since it was evident that specialist addiction service prescribers provided some level of support to them.

Furthermore, data collection was by a single researcher. There is the possibility that the researcher’s own perspectives may have affected interpretations that were made. However, the conduct, analysis and interpretation of data were overseen by two of the authors in addition to A.O.

## Conclusion

Assessment of the appropriateness of prescribed medications appeared to be a complex judgment. Optimal assessment of prescribing appropriateness should involve a balance between guideline recommendations, risks and benefits of prescribing, and the context. Nurse prescribers and medical doctors differed in their approach to reviewing medications but appeared to be working within their competency, with doctors providing support to nurse prescribers when needed. Prescribers were a sort of ‘safety net’ against medication-related risks to GPs. Recent changes in the UK drug and alcohol field have led to diminishing availability of medical expertise and an increasing reliance on non-medical prescribing. These changes have the potential to affect the quality of decision-making around medications. It appears there is a need to further empower non-medical prescribers and GPs to effectively manage service users with comorbidity.

## Electronic supplementary material

Below is the link to the electronic supplementary material.

Supplementary material 1 (DOCX 15 kb)

## Footnotes

1 Nurse prescribers had practiced in addiction specialty for between 1 and 5 years and as nurses for between 5 and 22 years. Two doctors had no prior experience in addiction specialty while the remaining had between 6 months and 35 years of experience. Consultant psychiatrists in particular, had between 3 and 35 years of experience in addiction specialty. Doctors had between 6 and 41 years of clinical experience.
