# Supplementary material for: Prescribers’ views and experiences of assessing the appropriateness of prescribed medications in a specialist addiction service
Source: Int J Clin Pharm. 2017 Oct 26;39(6):1248–55. doi: 10.1007/s11096-017-0541-4 (PMC5694531; doi:10.1007/s11096-017-0541-4)
Supplement: Supplementary file 1 — Supplementary material 1 (DOCX 15 kb) [file 11096_2017_541_MOESM1_ESM.docx]

TOPIC GUIDE FOR INTERVIEW WITH PRESCRIBERS

| The purpose of this interview is to explore your views on the appropriateness of prescribing for people with addiction problems. This interview should take between 30 and 60 mins. However, you can stop at any point if you do not want to continue.  With your permission, the interview will be audio recorded and transcribed verbatim.  Direct quotes may be used while writing up this thesis but these would be anonymous. Do you have any questions before we start this interview? |
| --- |

Topics to be explored

1. How would you define inappropriate prescribing?

[Prompts to be used where appropriate: use of guidance/policies when assessing prescribing, guidance/advice designed for this specialist addiction service, do you think inappropriate prescribing is a particular problem, and if so why].

1. What types of inappropriate prescribing do you encounter? Please describe them and the reasons why they are inappropriate.

[Prompts to be used where appropriate: who prescribed medications, what is inappropriate about them, potential reasons for such prescribing].

1. What classes of medications do you assess their appropriateness?
2. How do you assess the appropriateness of service users’ medications?

[Prompts to be used where appropriate: factors taken into consideration when assessing prescribing appropriateness, challenges encountered when assessing prescribing appropriateness, how often is appropriateness assessed, actions taken concerning inappropriate medications].

Is there anything else we have missed or should have talked about?

Thank you for your time.
